# Evaluation of an Electronic Medical Record Module for Nursing Documentation in Paediatric Palliative Care: Involvement of Nurses with a Think-Aloud Approach

**DOI:** 10.3390/ijerph19063637

**Published:** 2022-03-18

**Authors:** Sven Kernebeck, Theresa Sophie Busse, Chantal Jux, Larissa Alice Dreier, Dorothee Meyer, Daniel Zenz, Boris Zernikow, Jan Peter Ehlers

**Affiliations:** 1 Department of Didactics and Educational Research in Health Science, Faculty of Health, Witten/Herdecke University, 58448 Witten, Germany; theresa.busse@uni-wh.de (T.S.B.); chantal.jux@elisabethgruppe.de (C.J.); jan.ehlers@uni-wh.de (J.P.E.); 2 PedScience Research Institute, 45711 Datteln, Germany; l.dreier@pedscience.de (L.A.D.); d.meyer@pedscience.de (D.M.); b.zernikow@kinderklinik-datteln.de (B.Z.); 3 Department of Children’s Pain Therapy and Pediatric Palliative Care, Faculty of Health, School of Medicine, Witten/Herdecke University, 58448 Witten, Germany; 4 Smart-Q Softwaresystems GmbH, Lise-Meitner-Allee 4, 44801 Bochum, Germany; zenz@smart-q.de; 5 Pediatric Palliative Care Centre, Children’s and Adolescents’ Hospital, 45711 Datteln, Germany

**Keywords:** paediatric palliative care, participatory design, electronic health records, electronic medical records, technology acceptance, usability, user involvement

## Abstract

Background: Paediatric palliative care (PPC) is a noncurative approach to the care of children and adolescents with life-limiting and life-threatening illnesses. Electronic medical records (EMRs) play an important role in documenting such complex processes. Despite their benefits, they can introduce unintended consequences if future users are not involved in their development. Aim: The aim of this study was to evaluate the acceptance of a novel module for nursing documentation by nurses working in the context of PPC. Methods: An observational study employing concurrent think-aloud and semi-structured qualitative interviews were conducted with 11 nurses working in PPC. Based on the main determinants of the unified theory of acceptance and use of technology (UTAUT), data were analysed using qualitative content analysis. Results: The main determinants of UTAUT were found to potentially influence acceptance of the novel module. Participants perceived the module to be self-explanatory and intuitive. Some adaptations, such as the reduction of fragmentation in the display, the optimization of confusing mouseover fields, and the use of familiar nursing terminology, are reasonable ways of increasing software adoption. Conclusions: After adaptation of the modules based on the results, further evaluation with the participation of future users is required.

## 1. Introduction

Paediatric palliative care (PPC) (for acronyms see Appendix A) is a multidisciplinary and noncurative approach to the care of children and adolescents with life-limiting and life-threatening illnesses [1,2,3]. Approximately 21 million children are in need of PPC [4]. The conditions of patients undergoing PPC are often rare [5], complex, and range from neuromuscular [6] to genetic and congenital diseases [2]. In general, oncological diseases account for a smaller proportion of this total [7]. The aim of PPC is to provide psychosocial, physical, and spiritual support to children and their relatives [8], and to manage symptoms such as pain and impairment in communication [9]. Patients experiencing PPC undergo numerous admissions and discharges to and from inpatient and outpatient settings over the course of their lives [10]. To overcome the resulting fragmentation of health care systems, major challenges exist in the coordination of treatment and care [11].

The complex care and treatment of patients in PPC is reflected in high demands for documentation [12]. In particular, electronic medical records (EMRs) play an increasingly important role in PPC for documenting complex processes [11,12,13]. These records can facilitate communication among multiple professions [14] and support care coordination and planning [11]. In clinical practice, EMRs have numerous advantages in terms of providing different functions, such as access to patients’ data in real time, sharing information with other providers, and recording reminders and alerts [15]. EMRs can potentially increase the overall quality of care and time efficiency [16].

Nurses play an important role in PPC, as they are responsible for providing care and support for the affected patients and their relatives [17]. They are one of the major user groups for EMRs [18], and they spend a large proportion of their working time on documentation [19]. Although nurses generally express a positive attitude towards digital documentation [20], the introduction of EMRs can be accompanied by negative perceptions [21,22,23] and unintended consequences [24,25]. In the literature, consistent results are found for different countries [26,27,28]. For example, poor usability of EMRs has a negative impact on the wellbeing of nurses [29], and can produce stress and frustration [30] or cognitive failure [18]. Additionally, this factor is associated with burnout [28]. Similar results can also be identified for physicians as users of EMRs [31,32]. In different studies, nurses also tend to have a negative perception of spending more time on digital documentation than with patients [29].

To prevent such negative consequences of EMRs, it is necessary to involve nurses in the development of EMRs by using a participatory design process [33,34] to ensure that EMRs are context sensitive [35]. This process entails that EMRs and their functions must be adapted to the clinical context in which they are used. If this requirement is not considered, the implementation of EMRs can interrupt nurses’ usual ways of thinking and workflows, and thus lead to additional documentation work [21,36,37]. A participatory design process encompasses the involvement of users in a process of creative development, which is characterized by four main principles [34]: (1) democratization of decision-making processes; (2) mutual learning processes; (3) observation of latent (tacit) knowledge structures; and (4) mutual creativity through collaborative work among developers, researchers, and (future) users. This approach is a prerequisite to ensure that work processes and usability are considered during development [38] and remain a factor for the success of the implementation of EMRs [39].

It is relevant to apply a theoretical perspective of technology acceptance when carrying out participatory development for new technologies. Theories and models from acceptance research are used here, such as the “unified theory of acceptance and use of technology (UTAUT)” [40,41]. UTAUT includes four key direct determinants of behavioural intention to use a technology from the user perspective: performance expectancy, effort expectancy, social influence, and facilitating conditions [40,41]. In the context of acceptance research on EMRs, UTAUT is equally applicable [42,43].

This study is a part of the ELSA-PP project, which aims to adapt an EMR from adult care to inpatient settings that provide PPC. In this project, requirements for an EMR in the context of PPC were collected, and different modules were programmed [44]. For example, a novel patient chart module has already been evaluated with the participation of physicians and nurses [45].

Research question: How do potential future users in the context of inpatient PPC perceive the module for nursing documentation in terms of technology acceptance, and what are their wishes for improvement of the module?

The aim of this study was to evaluate the acceptance of a module for nursing documentation for the inpatient setting of a PPC unit from the perspective of potential users in the context of PPC and to involve those users in the development process.

## 2. Materials and Methods

### 2.1. Study Design

For the evaluation of the module for nursing documentation, a qualitative observational study was conducted in September 2021 over a period of three weeks. The method of concurrent thinking aloud (CTA) was combined with a subsequent qualitative interview [46]. With CTA, cognitive processes and reasons for user actions can be assessed while interacting with a technology [47]. In the context of a task-oriented approach, participants were asked to perform everyday tasks of nursing documentation using the novel module and to verbalize their thoughts while doing so [46,48]. In usability testing of new technologies, CTA is the most frequently used method [49], and it is also used in participatory design [33].

The ethics committee of Witten/Herdecke University obtained ethical approval for this study (approval code: 35/2019).

### 2.2. Participants and Recruitment

Nurses from a PPC unit of a children’s and adolescents’ hospital in Germany were asked to participate in this study. Posters and flyers were distributed to provide information about the study’s aim and procedure. In addition, reminders were given during shift changes at the PPC unit. Further written informational material and a consent form were handed out if nurses expressed an interest in participating. Financial compensation of €40 for taking part in the study was offered. At this PPC unit, the participants are documenting the majority of their clinical documentation in paper-based records.

### 2.3. Data collection and Testing Procedure

To ensure as much participant flexibility as possible, two options were provided for the testing procedure: (1) remote and (2) face-to-face. In both options, two researchers were present at almost every session, with one conducting the interview and one writing field notes. The procedure for data collection was otherwise identical for both options. Audio and screen movements were recorded as a screencast using the software Captura (version 8.0) in both cases.

(1) Remote testing: During remote sessions, participants shared their screen with evaluators using the software Zoom. Relevant documents were sent to participants in advance by email.
(2) Face-to-face testing: Face-to-face testing sessions were conducted in a regular office close to the PPC unit, with the evaluators using a standardized software and hardware setup. The hardware setup consisted of a computer with a mirrored screen so that the researchers could observe the use of the modules.

At the beginning of each session, participants were informed about the aims and methodical procedure of the study. From previous studies in the ELSA-PP project, most of the nurses in the PPC department of the children’s and adolescents’ hospital were already familiar with the procedure [45,50]. Within the task-oriented framework, the tasks served to structure the CTA and were presented in printed form. Participants were instructed first to read all tasks aloud and were encouraged to verbalize all thoughts while performing the tasks. Participants were informed that the researcher would remind them to think-aloud if they ceased verbalizing their thoughts [46]. This approach is recommended when using the CTA method, because participants reducing or completely ceasing verbalization after some time is a known behaviour [51]. To reduce discomfort among participants, they were told that there was “no right or wrong” in testing, and that the aim of the study was not to test them, but to test the module [51]. The modules were filled with exemplary dummy data concerning a fictional patient to stimulate the thinking of the participants.

For this study, after the completion of the tasks, an exploratory and open approach was chosen to discuss solutions with the participants in the spirit of participatory design [49]. Nevertheless, during the performance of the tasks and the duration of thinking aloud, interaction with the participants was kept to a minimum to reduce bias [46]. Participants were advised that if they encountered problems when using the modules, they should first look for a solution themselves. However, they could ask for help at any time. For this purpose, standardized questions for problem solving were formulated to reduce bias due to the influence of the researchers (Appendix B) [51].

A semi-structured interview guide was developed through discussion and a final process of consensus building by the researchers. The questions were related to general impressions of using the modules as well as to aspects of usability, comprehensibility, and future wishes for further development (Appendix C).

### 2.4. Module for Nursing Documentation

**Figure 1:**
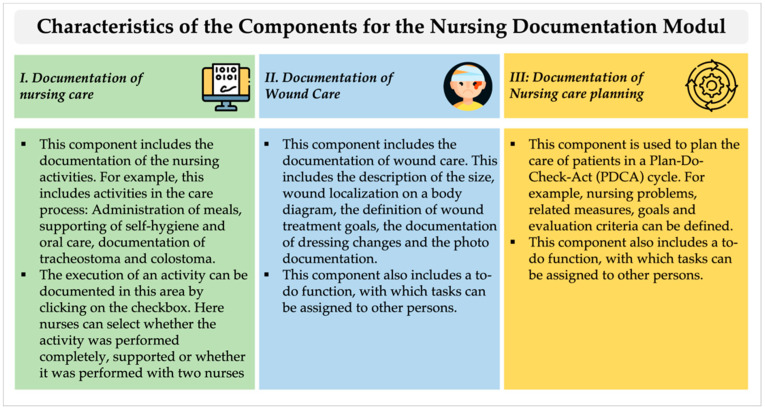
Overview of the characteristics of components for the nursing documentation module. (Icons made by Freepik from www.flaticon.com, accessed on 28 November 2021).

The module for nursing documentation consists of three components in which nursing activities can be documented: (I) nursing care, (II) wound care, and (III) nursing care planning (Figure 1). The components were developed as part of the ELSA-PP project and were integrated into the software Information-System Palliative Care (ISPC) (company: smart-Q, Germany), which is used for adult palliative care. The module does not include certain aspects of documentation, such as vital signs, symptoms, or wake−sleep rhythm, which were the subject of a previous study in which a patient chart module was evaluated [45].

### 2.5. Data Analysis

Audio files were transcribed verbatim following the simple rules developed by Dresing and Pehl, and were transferred to MAXQDA (2020) alongside related videos [52]. The analysis was carried out independently by two researchers (S.K. and T.S.B.). First, all statements and observations were assigned to the tasks and were annotated with the relevant screen movements. Subsequently, these statements and observations were deductively assigned to the central determinants of the UTAUT, which serve as the main category (Table 1). Subsequently, inductive subcategories were formed. This process resulted in a coding system, which was finalized through discussion and consensus building (L.A.D. and C.J.). Quotations were translated into English and assigned a pseudonym (ID) (Nurse_transcript-ID_#timecode#).

## 3. Results

Testing sessions were conducted with eleven female nurses (Table 2), including seven face-to-face sessions and four remote sessions. Session duration ranged from 43 min to 97 min, with an average of 56 min.

**Figure 2:**
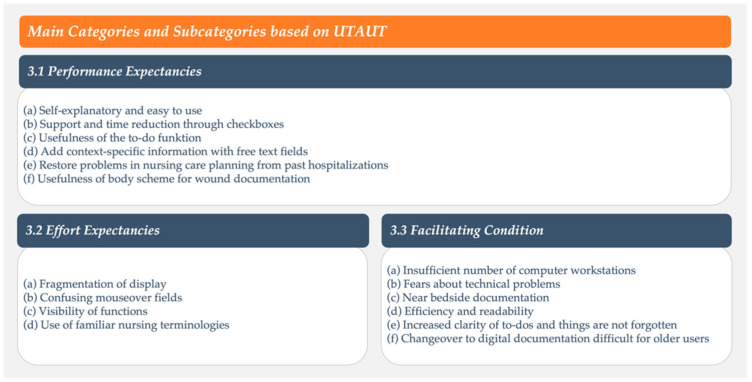
Overview of the main categories and subcategories based on UTAUT.

In total, 499 codes were assigned within the coding system, with a range of 35 to 60 codes and an average of 45 codes per interview. Due to the large number of observations and statements, only particularly significant categories with the largest number of codes are reported below. Three main categories were formed, (I) performance expectancies, (II) effort expectancies, and (III) facilitating conditions, with a total of 16 subcategories (Figure 2). No codes could be assigned to the UTAUT determinant social influence.

### 3.1. Performance Expectancies

This theme includes all topics regarding “the degree to which an individual believes that using the system will help him or her to attain gains in job performance” [41], which encompasses mainly the functionalities of a technology based on UTAUT.

- (a) Self-explanatory and easy-to-use

As a general impression, participants perceived all three modules as self-explanatory and easy-to-use. This impression was often complemented by verbalizations that they basically had to become accustomed to the application.

“Well, in itself I think it is quite good and also as detailed as it is now described here, always quite self-explanatory. But I think it is still quite difficult at first, because it is simply a change, and at the beginning, we will just click on different things, I think, to look for where it is really to be found in the end or where I can enter something exactly again.”

(Nurse_01_#01:12:15#)

- (b) Support and time reduction through checkboxes

Participants perceived the use of checkboxes in all components to be practical and useful. This feature was particularly discussed by many participants in the documentation of wound care, as the documentation of wounds was often perceived to be time-consuming. Here, examples were highlighted when complex wounds needed to be treated, such as in patients with epidermolysis bullosa.

“But I think if you really have such patients [with decubiti], or we just have a patient with, for example, epidermolysis bullosa, where you also have to change the dressing every two days, it is quite practical if you just have to mark a [predefined] list with a cross [the checkbox] concerning what the wound is cleaned with and what it is fixed with and what kind of materials are used. Because otherwise, it is a lot of work to keep lists and write things down.”

(Nurse_06_#00:29:23#)

- (c) Usefulness of the to-do function

In general, the to-do function in the components concerning wound care and nursing care planning were perceived to be useful in supporting nursing care. Here, the ability to define tasks for the nurse’s own work, as well as assigning tasks to other colleagues, was described as useful. The use of this function was also seen as a means of facilitating communication with other professions, e.g., when working with physicians.

In addition, it was desired in this context that the to-do lists could be grouped individually. For example, if a nurse is looking after three patients, it should be possible to display only the to-do lists for these patients.

“So, I think that is really good. And that I can then perhaps also select times, so, I would like to select that a physician is coming to the next care. Because the doctors would then probably also see that it is a to-do for them. And I do not have to run after them and say, ‘Remember, you have to come to change the dressings or something else’. I think that is definitely very, very good.”

(Nurse_01_#00:51:43#)

- (d) Adding context-specific information with free text fields

In all three components, participants requested the ability to enter contextual information by adding free text fields. For example, in the documentation of nursing activities, a free text field was requested to add information concerning the specific materials that were used in nursing care. Furthermore, a free text field was requested for the description of wounds, as there were only checkboxes with predefined terms for the description of wounds.

Nurse: “Can I add additional information somewhere, so that everyone [colleagues] also knows, aha, the skin must be creamed with the cream XY and not with wound protection cream?”.

Interviewer: “Is it not intended yet, but you would just wish that one could insert information here as free text?”.

B: “Yes, for example. We often write additional information in the [paper-based] nursing documentation, and that corresponds a bit to the care planning here. That you then see: Aha, we are now using cream XY or water only. I would miss that here now.” (Nurse_09_#00:10:51#).

- (e) Restoration of problems in nursing care planning from past hospitalizations

When documenting the nursing care plan, participants requested that problems from past hospitalizations be restored from the archive as current problems. The rationale for this request was that patients undergoing PPC are often readmitted as the disease progresses, and the problems of such patients are often seen again in the context of new hospitalizations. Here, it was considered a work-saving measure for problems not to have to be redefined and re-described in their entirety.

“Yes, I would like that. So, if the nursing problems are transferred to the archive, if the patient was already here in May [in the PPC unit], I say, and the patient comes back now and I can actually take over half of the problems one to one, because it has not changed. For example, if the patient still tends towards constipation or still has secretion problems, you still do the same thing, that you can quite simply just click, and it [the software] takes over automatically.”

(Nurse_08_#01:19:08#)

- (f) Usefulness of the body scheme for wound documentation

The participants particularly emphasized that they found the body scheme helpful in marking wounds. They often described this process as being more precise and quicker to draw than the performance of the same task on paper.

“Oh yes, it works. Exactly, I think it is good that you have such a scheme, such a body scheme. Because sometimes you cannot describe it as well as you might have seen it, i.e., where the spot is. So, I think that is great. Exactly, here I can enter everything with five centimetres, two centimetres, whatever.”

(Nurse_08_#00:32:25-7#)

### 3.2. Effort Expectancies

This theme includes all topics regarding “the degree of ease associated with the use of the system” [41], and basically includes the dimension of perceived usability and complexity of use.

- (a) Fragmentation of the display

**Figure 3:**
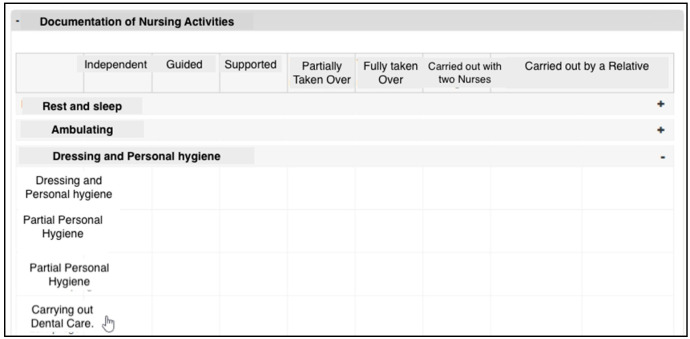
Documentation of the component for documenting nursing activities.

The participants perceived a fragmentation of the display in the component for documenting nursing activities. When documenting nursing activities, the fact that the heading (Headline in Figure 3) was moved out of the field when scrolling down was found to be impractical.

“What I find a bit impractical when I scroll down is that you then no longer see the headline: then have to scroll up again and then I see which nursing activity I have to click on. And where did I end up now?”

(Nurse_08_#00:08:29#)

- (b) Visibility of functions

In the component for documenting nursing activities, participants were not able to identify how nursing activities should be documented. This issue was attributed to the fact that the checkboxes that had to be clicked to document the nursing activities were not visually raised. The checkboxes were greyed out, as were the headings. Therefore, a large percentage of participants became irritated, which led to a longer search for the function (checkboxes).

“If I click on that now? Can you do that? No. […] But you cannot click on that now? […] Well, this is all here not to click on, no. I find that irritating now, because I do not know how to do document the nursing activities.”

(Nurse_03_#00:04:05#)

- (c) Use of familiar nursing terminology

In the component for documenting nursing activities, the free text field for documenting the nursing report was not recognized as such by the participants. This issue was particularly the result of the fact that the free text field was labelled “comment” and did not use the specific phrase “nursing report”; nevertheless, some participants saw and named the field comment directly.

Nurse: “Okay, in the nursing report, in the free text, I have to document a nursing report. It is up there now. Just looking. […]. Cannot add it here, can I? Oh, maybe in the filed ‘comments’”.

Interviewer: “But I could add some free text there now. But you would not have recognized it as such now, so to speak?”.

Nurse: “I would not have recognized it [the field ‘comments’ for ‘nursing report’]”.

Interviewer: “What would help you recognize that sooner?”.

Nurse: “I think that would really have to be called nursing report too.” (Nurse_03_#00:09:38#).

- (d) Confusing mouseover field

**Figure 4:**
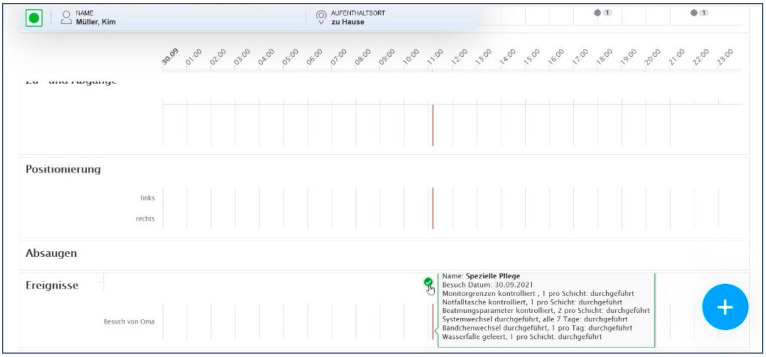
Mouseover field for documenting nursing activities in the patient chart view.

For nurses to be able to track which activities have been documented, all documentations are transferred to the patient chart view (Figure 4). The patient chart view is the centre of the EMR, where physicians and nurses document, e.g., vital signs, sleep patterns, symptom observations, or notes on catheter management. When presenting documented nursing activities in the patient chart view, the mouseover field in which the activities were presented after saving the documentation was perceived to be confusing (Figure 4).

“I find that [the mouseover field] totally confusing here. If I click on it now, it shows me what I have done [which activities were documented]. I find that super confusing. I wouldnot be able to cope with it, I would give up again.”

(Nurse_09_#00:32:08#)

### 3.3. Facilitating Conditions

This theme includes all themes regarding “the degree to which an individual believes that an organizational and technical infrastructure exists to support use of the system” [41].

- (a) Insufficient number of computer workstations

Concerns were described that after the implementation of digital documentation with EMR, an insufficient number of computer workstations would be available. This situation could create conflicts, because not all nurses would be able to document at the same time.

“That we do not have enough workstations with computers. That one wants it, the next and there, that there is friction, exactly. Yes, that would be one of the biggest fears I have.”

(Nurse_11_#00:46:12#)

- (b) Fears concerning technical problems

Fears concerning technical problems were frequently expressed. In particular, fears concerning losing an internet connection or a general system crash were described. In particular, concerns were described about not being able to access patient information, such as information regarding medications, in an emergency.

“That it does not work and I cannot access the electronic medical record. That would give me a stomach-ache, that I just could not get to the things that were important for the patient right now. That I do not know, okay, now I do no know, the patient is cramping, I need a medication now, but I do not know what I can give because I cannot access the electronic medical record. Or what if the internet time is not working or the connection is just too slow.”

(Nurse_09_#01:30:08#)

- (c) Near-bedside documentation

In addition, from the participants’ perspective, it would be useful if documentation could be carried out near the bedside of the patient. This location for documentation was perceived to be easier because the nurses do not have to remember the contents of the nursing documentation or have to write them down on a piece of paper by hand. In the latter case, they have to document this information at a workstation away from the bedside.

“But otherwise with the implementation [of the EMR], is it meant that we then have it [the EMR] on a tablet, so we could take it with us [to the patient’s room]? Because I think that if you take it with you, you can do it directly after you have done something [e.g., nursing activities] with the child. Or whether you must go back [to a workstation] again first.”

(Nurse_08_#00:56:09#)

- (d) Efficiency and readability

In addition, participants expressed the hope that digital documentation would increase the efficiency of nurses’ work by eliminating the burden of handwritten documentation. In this context, it was also noted that readability is increased by digital documentation, as handwriting is often difficult to read.

“I am hoping that once you are well into it [the digital documentation] and you know where to enter what, that it will not take as much time as the handwritten documentation. That, and maybe also that you do not have to write anymore so much [by hand]. That it is all easy to read, above all, even the doctor’s handwriting. And that it is partly also clearly shorter, more compact […].”

(Nurse_03_#01:00:15#)

- (e) Increased clarity of to-do lists and the assurance that things are not forgotten

Moreover, the hope was mentioned that EMR would make it less likely for nursing activities to be forgotten, as the EMR would provide a to-do function. As such, EMR provides automated reminders of what nursing activities still need to be completed.

“In the long run, I hope that it will make my work easier […]. That it is clear what you have to do. That the to-dos pop up in such a way that I also forget fewer things in the end or simply that fewer things get lost. Maybe also a little bit that things that are sometimes considered unnecessary in certain situations, that they are then extra penetratingly annoying with the to-do pop-ups so that you just do it and you do not forget it anymore.”

(Nurse_01_#01:00:15#)

- (f) Changeover to digital documentation difficult for older users

From the participants’ point of view, the transition to digital documentation would be more difficult for older nurses than for younger nurses (e.g., due to low digital competencies). However, participants were also confident that older caregivers would be able to cope with the transition after a period of familiarization.

“I think that [change to] digital documentation will be more difficult for some older colleagues, but I think that if you put your mind to it a bit and you get used to it, they would be able to do it quite well, and I have to be prepared for it.”

(Nurse_05_#00:46:17#)

## 4. Discussion

The aim of this study was to evaluate the acceptance of a module for nursing documentation for the inpatient setting of a PPC unit from the perspective of potential users involved in PPC, and to involve those users in the development process.

In summary, participants perceived the module to be self-explanatory and intuitive. In particular, a reduction in documentation workload was frequently associated with the application of the module in EMR. Based on the results identified in this study, themes are discussed that can be assessed as particularly relevant for future design and further development of the module and the EMR itself.

In the main category of performance expectancies, various functions were identified that were considered to be particularly helpful to the participants in clinical work.

In this study, assigning tasks with the to-do function was perceived as useful, especially in the components focused on wound care and nursing care planning. In addition to making work easier and reminding nurses of tasks to be completed, this function was also seen as helpful for interprofessional collaboration with physicians. According to the current literature, one of the key functions of EMR for nurses is to communicate with other professionals and to structure patient care [37]. For this purpose, auxiliary functions such as instant messaging and task management software can support interprofessional collaboration [53].

Even though the to-do function has been perceived as helpful, its application also poses dangers, which are a risk in clinical practice. If such functions are used, for example, to assign tasks to physicians, those physicians will receive a message for each task assigned. Consequently, the processing of such electronic messages must be done by physicians, which can lead to an additional burden on top of numerous other messages [54]. The processing of such electronic messages poses the risk of an additional burden for physicians, who are known to bear major burdens [55]. This situation can lead to work-related frustration. One study suggested that the management of messages may be linked to the development of burnout in physicians [56]. For this reason, the use of this function should be planned and coordinated interprofessionally, both during further development and during implementation.

Moreover, in this study and in other studies, one of the main perceived benefits of using EMRs was an increase in work efficiency, as EMR use was viewed as saving time spent on documentation [14,57]. In this study, the use of checkboxes and defined lists was found to be particularly helpful and to make the work of caregivers easier. Although, on the one hand, the use of such features seems useful, such use is also accompanied by concerns. For example, in a qualitative study of nurses’ views on the impact of EMRs, the use of checkboxes and lists was viewed critically [58]. Nurses in that study expressed concern that the use of checkboxes, predefined dropdown menus, or cut-and-paste functions limited nurses’ critical thinking and that nurses who used such functions would unlearn how to think “outside the box”.

One way of documenting content “outside the box” is to use free text fields. This functionality was requested by many participants in this study in order to add contextual information to specific content documented using predefined lists and checkboxes. Although adding free text is important to be able to document specific content, using free text fields too often can also be dangerous. For example, one study found that the use of free text fields led to a kind of “data overflow” among users due to the large amount of text to be read [59]. It should therefore be carefully considered what sorts of content makes it necessary to use free text fields, and it is also important to identify the situations in which it is necessary to use checkboxes or predefined lists. This dilemma should be considered when implementing EMR in the context of PPC prior to implementation, and requires further critical reflection hereafter.

In the main category of effort expectancies, it was also possible to identify the factors that play a special role in the further development and adaptation of EMRs to positively influence acceptance.

In particular, the fragmentation of the display in the component of nursing documentation was perceived by nurses as an area in need of improvement. Fragmentation of the display is a phenomenon that is often found in the use of EMRs [48]. The fragmentation of the display leads to users having to memorize content and consequently requires them to scroll back and forth to bring information together. In addition to general workload, this difficulty also places an additional mental load on the user [32]. This sociotechnical variable is also referred to as cognitive load [60]. Therefore, one strategy for optimizing the modules is to reduce fragmentation of the display. For example, a freeze function in the module for documenting nursing activities could be used in this case to make a heading visible in the display at all times, so that users do not have to remember the contents of this heading.

The facilitating conditions identified here potentially contribute to the development of an implementation strategy for the EMR module in the context of PPC. For example, an insufficient number of computer workstations was a concern highlighted by participants. Consistent with the evidence, limited access to or a limited number of computers is frequently mentioned as a barrier to the implementation of EMRs in general [14]. Therefore, for possible implementation, the concrete requirements of hardware equipment must be considered.

Likewise, it is important to address fears concerning technical problems prior to implementation. In this context, nurses and physicians, as well as other users, need to be trained to deal with technical problems in clinical practice. For example, questions need to be answered concerning how technical support can be provided in an emergency. Insufficient technical support is also highlighted by a scoping review on barriers to the adoption and use of EMRs [14].

Furthermore, concerns were voiced by participants regarding how older nurses would manage the transition from handwritten to digital documentation due to their potentially low digital competencies. These concerns are consistent with evidence from other studies on the effects of the implementation and use of EMRs for older nurses compared to their younger counterparts [21]. For example, a study conducted in an intensive care unit found that nurses’ age was negatively associated with perceptions that the use of EMRs interfered with the direct care of patients and did not increase work efficiency [29]. Here, older nurses expressed their belief that the time spent on digital documentation was inadequate compared to time spent on patient care [29]. Therefore, learning conditions and learning needs must be adapted to these user groups. The content of the training must be oriented to their daily work processes and must include the ability to learn on the job. Similarly, digital training and learning environments should be provided [61]. However, insufficient training for the use of EMRs is considered to be a major barrier for EMR adoption in general [14]. Therefore, regardless of specific training concepts for older users, sufficient training of all users is also necessary, which can increase the quality of documentation [62].

It is also relevant to interpret the results of this study from a sociotechnical perspective. For example, care in the context of PPCs is multidisciplinary in nature [8]. Thus, under everyday clinical conditions, EMRs are used by nurses in interactions with other professional groups, such as physicians and social workers. A recent review of the effects of the implementation of EMRs has shown that EMRs can both improve and worsen communication [14]. Consequently, the impact of the modules evaluated here on communication with other health professionals in the context of PPC should be considered by further steps of the evaluation.

For the further development of the modules and the EMR itself, it is necessary to first implement the factors identified here, which can potentially influence acceptance. Subsequently, these adjustments need to be re-evaluated by conducting further iterations with users. Following this re-evaluation, consideration should also be given to the task of complementing the evaluation by other methodological approaches that are suitable for later phases of the EMR development process. Here, after sufficient consideration of users’ needs, expert-based methods such as heuristic evaluation or cognitive walkthroughs should be applied [48]. Other methodological approaches that simulate clinical situations should also be considered, such as “near live clinical simulations” (NLCS), in which actors pretend to care for real patients so as to provide a more realistic application scenario for evaluation [63].

In the interpretation of results, it should be considered that in this study, only individual modules of EMR were evaluated. Under clinical conditions, the application takes place while nurses interact with multiple modules and functions of EMRs [45]. Future evaluations should ensure that interaction with additional modules of EMR is evaluated.

Furthermore, the sample in the study could be considered sufficient to achieve data saturation. In this context, there is some discussion in the literature concerning the required sample size, especially when analysing the usability of new technologies. Drawing on the literature concerning usability analysis, 80% of all usability problems can be identified by five users [64]. In general, it is recommended to perform several iteration cycles with fewer participants instead of testing many users directly [65].

Despite the methodological limitations inherent to the application of the CTA method, the following points must be noted. The CTA method makes it possible to analyse and assess the cognitive processes of users during the execution of tasks, which provides valuable insights into users’ mental models [66]. Furthermore, user actions and reactions, as well as verbalizations, can be assessed and recorded simultaneously, which provides valuable and rich data for the redesign of technologies according to users’ needs. When the formative evaluations of the modules are completed, quantitative evaluation procedures should be used to focus specifically on the analysis of usability and task performance. Here, a retrospective think-aloud approach is recommended [67].

## 5. Limitations

The results obtained here must be assessed in light of the applied methodology [47]. During the use of the CTA method, the presence of observational evaluators may have influenced participant behaviour and verbalization (the Hawthorne Effect) [68]. It can be assumed that some participants may have formulated their statements more positively or suppressed negative statements due to social desirability [63]. Nevertheless, it cannot be excluded that the results, respectively the behaviour and the statements of the participants, are biased due to social desirability [64].

Moreover, in addition to the application of the software itself, the CTA method also affects the cognitive load of participants, as they must concentrate on performing tasks and verbalizing simultaneously [47]. The resulting dual cognitive load may have influenced the completeness of the verbalized information [68]. This factor probably also influenced the participants’ perceptions of their cognitive load after the application of the modules. It is also hypothesized that participants’ simultaneous focus on task performance and verbalization may lead to incomplete data [69]. This incompleteness results from the fact that, due to cognitive focusing, participants forget to report other important information.

Moreover, it must be considered that CTA was applied under laboratory conditions during a short application period. It must be assumed that the results may differ in everyday practice under stressful clinical situations [65]. In addition, it is likely that further needs will be verbalized by users during longer-term use of the modules in clinical practice. This fact must be taken into consideration given the sociotechnical nature of EMRs. In general, EMRs are not considered static [70], nor are hospitals or the knowledge and competences of nurses [65]. This situation means that because of the dynamic sociotechnical environment of health care, continuous adaptation of the software will be required. This requirement applies even after further iterations and even after the implementation of the evaluated modules [71]. As a natural consequence, on the one hand, the results presented provide a profound basis for understanding potential factors concerning acceptance and the general needs of digital documentation in PPC from the perspective of nurses. On the other hand, those elements change over time and require user-driven optimization [72].

Finally, it must be mentioned that the results obtained here relate to the context of use in the German health care system. It is likely that in different countries, other requirements are relevant.

## 6. Conclusions

This study identified factors that may have a positive impact on user acceptance. These results were obtained by effectively involving potential users in the development process. Overall, participants perceived the novel modules for nursing documentation as intuitive and self-explanatory.

In further development, the wishes of users should be implemented, and special attention should be given to reducing information overload and display fragmentation. Further iterations of the evaluation should be conducted with users by simulating real clinical conditions.

## Figures and Tables

**Table 1:** Definitions of the core direct determinants of UTAUT [41].

UTAUT Determinant	Definition
Performance expectancy	“The degree to which an individual believes that using the system will help him or her to attain gains in job performance,” which encompasses mainly the functionalities of a technology.
Effort expectancy	“The degree of ease associated with the use of the system,” which basically includes the dimension of perceived usability and complexity of use.
Social influence	“The degree to which an individual perceives that important others believe he or she should use the new system”.
Facilitating conditions	“The degree to which an individual believes that an organizational and technical infrastructure exists to support use of the system”.

**Table 2:** Overview of participant characteristics.

Characteristic	*n* (%)
Sex	
Female	11
Male	0
Age in years (Mean)	36 ^1^
Profession	
Nurse	11 ^1^
Years of PPC experience	
0–9	3 ^1^
10–20	4 ^1^
>20	2 ^1^
Years of experience in current position	
0–9	4 ^1^
10–20	4 ^1^
>20	1 ^1^
Experience in professional use of EMR	0 ^1^

^1^ Characteristics from one participant are missing due to the non-return of the questionnaire.

## Data Availability

The corresponding datasets are available from the corresponding author upon reasonable request.

## Appendix A. Acronyms

PPC	Paediatric palliative care
UTAUT	Unified theory of acceptance and use of technology
EMRs	Electronic medical records
CTA	Concurrent thinking aloud
ISPC	Information-system palliative care
NLCS	Near live clinical simulations

## Appendix B. Interview Guide

Questions during the session to solve problems:

– Can you describe to me in your own words the problem you are having right now?
– What would you expect here?
– What would you have expected here (instead)?
– How would you solve the problem?
– What would you like to see happen?

Per module: What are the specifics that should/must be added?

## Appendix C. Guide for the Qualitative Interview

1. How would you describe your overall impression of the application?
2. Nursing documentation in general: What else would you like to see in the EMR regarding nursing documentation in the context of PPC?
  (a) Is there anything that you felt was missing?
  (b) Is there anything that you did not feel was necessary?
  (c) What other adjustments would you like to see?
3. If not yet named: How did you find the service?
  (a) Is there anything that you liked about it?
  (b) Is there anything that you did not like about it?
4. Can you describe in your own words how you would rate the clarity of the new modules: Was the content easy/quick to find?
5. If you imagine that the EMR/digital documentation were introduced in practice for your unit:
  (a) Can you describe for me in your own words what your fears would be if there was a switch from analogue to digital documentation?
  (b) Can you describe to me in your own words what benefits you would hope to gain if there were a switch from analogue to digital documentation?
6. How did you feel about the test situation?
  (a) ... especially the thinking out loud and the general situation?
